# Bacterial Communities in the Alpaca Gastrointestinal Tract Vary With Diet and Body Site

**DOI:** 10.3389/fmicb.2018.03334

**Published:** 2019-01-18

**Authors:** Courtney Carroll, Kyle D. Olsen, Nathan J. Ricks, Kimberly A. Dill-McFarland, Garret Suen, Todd F. Robinson, John M. Chaston

**Affiliations:** ^1^Department of Plant and Wildlife Sciences, Brigham Young University, Provo, UT, United States; ^2^Department of Bacteriology, University of Wisconsin–Madison, Madison, WI, United States

**Keywords:** microbiota, alpaca, camelid, gastrointestinal, forages

## Abstract

Gut -associated microbes (‘gut microbiota’) impact the nutrition of their hosts, especially in ruminants and pseudoruminants that consume high-cellulose diets. Examples include the pseudoruminant alpaca. To better understand how body site and diet influence the alpaca microbiota, we performed three 16S rRNA gene surveys. First, we surveyed the compartment 1 (C1), duodenum, jejunum, ileum, cecum, and large intestine (LI) of alpacas fed a grass hay (GH; tall fescue) or alfalfa hay (AH) diet for 30 days. Second, we performed a C1 survey of alpacas fed a series of 2-week mixed grass hay (MGH) diets supplemented with ∼25% dry weight barley, quinoa, amaranth, or soybean meal. Third, we examined the microbial differences of alpacas with normal versus poor body condition. Samples from GH- and AH-fed alpacas grouped by diet and body site but none of the four supplements significantly altered C1 microbiota composition, relative to each other, and none of the OTUs were differentially abundant between alpacas with normal versus poor body conditions. Taken together, the findings of a diet- and body-site specific alpaca microbiota are consistent with previous findings in ruminants and other mammals, but we provide no evidence to link changes in alpaca body condition with variation in microbiota relative abundance or identity.

## Introduction

Ruminants and camelids rely on the microbes in their gastrointestinal (GI) tracts to access energy and nutrients from the plant material they consume. For example in the alpaca (*Vicugna pacos*), a member of the camelid family, good body condition (GBC; Pryce et al., 2001) is an indication of low stress (i.e., environmental, nutritional or disease) and a gauge for overall health and survival. GBC is a specific index of the alpaca’s energy balance, reporting the subcutaneous fat that is the last fat deposit accrued during positive energy balance and the first to be mobilized during negative energy balance (Rastani et al., 2001). Alternatively, low body condition (LBC) or negative energy balance can be associated with low productivity of the herd (Reyna, 2005; Kristjanson et al., 2007). Further, we have observed that even within a well-fed alpaca herd, a number of animals tend to exhibit chronic LBC despite efforts to treat all animals equally with respect to deworming, vaccination, and access to feed and water. In these chronic LBC animals, body condition also appears to be independent of foraging success, such as time spent grazing or amount of diet consumed, and social hierarchy, where dominant animals have feeding priority over other members of the herd. Therefore, an explanation for this chronic LBC is still lacking.

The microbiota contributes to energy balance in ruminants (Shabat et al., 2016), and we hypothesized variation in the microbiota could be a characteristic that is linked to alpaca body condition in chronic LBC animals. Unlike true ruminants, which make use of a four-chambered forestomach, the camelid family is classified as a pseudo-ruminant and possesses a three-compartment forestomach. The first two compartments, of which the first (C1) comprises most of the volume, function similarly to the rumen/reticulum and omasum of true ruminants (Vallenas et al., 1971). Due to their greater feed retention time (San Martin and Bryant, 1989), increased microbial yield, and presence of glandular saccules in the forestomach, camelids possess a higher efficiency of fiber degradation when compared with ruminants, particularly when fed low-quality, low-protein forages (Rübsamen and von Engelhardt, 1979; Genin and Tichit, 1997). Volatile fatty acids (VFAs) – primarily acetic, propionic, and butyric acid – are released as by-products of microbial fermentation in the C1 (Stevens et al., 1980), and are used by camelids as a major energy source (Bergman, 1990). As with ruminants, pseudo-ruminants require glucose to meet the energy needs of specific tissues (brain, placenta), in which propionic acid is shuttled through the gluconeogenic pathway to produce the needed glucose (Bergman, 1990). For other tissues, acetic and butyric acid are the predominant energy precursor (Bergman, 1990). These findings underscore the importance of the digestive tract – especially the C1 – in maintaining proper energy balance in the alpaca. The microbiota of the alpaca digestive tract has only been defined previously in the foregut (Pei et al., 2010, 2013; St-Pierre and Wright, 2012; Henderson et al., 2015), and there are no direct links between variation in the microbiota and alpaca body condition.

The basis for this study is that established links between the microbiota and ruminant energy balance (Wang et al., 2012) suggest that the alpaca microbiota may potentially influence these processes. Since little is known about the composition of the alpaca microbiota, aside from in the forestomach, or how the alpaca microbiota responds to dietary perturbations (Pei et al., 2010, 2013; St-Pierre and Wright, 2012; Henderson et al., 2015), we took a three-fold approach. First, we defined the alpaca microbiota in six digestive tract sites and the response in each site to dietary forage (grass or alfalfa) variation (5 alpacas per treatment). Second, we examined the influence of minor dietary variation on the alpaca microbiota by surveying the C1 of a second alpaca cohort (4 alpacas total) all fed the same grass forage diet as in the first approach supplemented with different natural grains. Finally, we tested the prediction that the C1 microbiota differs between LBC and GBC alpacas (18 alpacas). Our results showed substantial variation in the microbiota with both body site and two different forage diets, but not when animals were fed a standard diet with additional grain supplements. Also, there were no significant differences in microbiota composition between alpacas with chronic differences in body condition. This last result suggests that variation in identity and abundance of the microbiota may not be a key determinant of alpaca body condition.

## Materials and Methods

### Sample Collection

Three experiments were designed in accordance with Animal Care and Use Guidelines (McGlone et al., 2010) and with approval of The Camelid Center and the Brigham Young University (BYU) Animal Use Committee (#16-1104). Within each experiment, alpacas were treated equally in regards to environment and feed administration. Digesta samples were taken upon completion of each experiment. All samples within an experiment were taken at the same time and from the same location within each organ, unless otherwise noted.

#### Forage Diet Experiment (Experiment 1)

In a previous experiment (Oldham et al., 2014), ten adult male alpacas (3+ years old; 65 ± 4 kg body weight [BW]) were divided into two groups. Each group was fed a different diet [grass hay (GH; tall fescue, *Festuca arundinacea*) or alfalfa hay (AH; *Medicago sativa*) for 30 days; chemical composition of the diet in these animals was reported previously (Oldham et al., 2014)]. The alpacas were housed in drylot paddocks and fed once daily *ad libitum.* Dry matter intake was determined by subtracting the weight of refused feed from the weight of administered feed on a daily basis, as described previously (Oldham et al., 2014). Briefly, the daily diet was fed at 10% above the amount consumed the previous day. Refused feed was collected the next day, dried at 60°C for 24 h, and weighed. As reported previously, average group-level daily dry matter intake was 1450 and 1375 g/day for AH- and GH- fed alpacas, respectively (Oldham et al., 2014). Alpacas were also provided with water and a commercial free-choice salt and mineral supplement *ad libitum.* At the end of 30 days the alpacas were sacrificed at a commercial slaughtering facility 2 h post-feeding. To facilitate sampling, one alpaca from each treatment was sacrificed on a given day, over a 5-day period. The digestive tract was immediately removed and divided into compartments 1–3, duodenum, jejunum, ileum, cecum, and large intestine. Digesta samples were taken from compartment 1 and from each of the intestinal subsections. For the C1, a total digesta sample was removed by hand and the liquid portion was obtained by squeezing by hand the liquid from the solid material into a sterile sampling container. The first 45 cm of the small intestine were assigned as the duodenal section and the remaining small intestine was divided in half into the jejunum and ileum, and samples were collected from 10 cm sections in the middle of each site. The cecum was evacuated into a sterile container by hand, homogenized, and subsampled. The large intestine sample was collected 40 cm from the rectal sphincter. The samples collected from each site contained both liquid and plant particulate matter. They were placed in a sterile container and immediately stored at −20°C. For DNA extraction, the samples were thawed on ice, homogenized, and 250 mg were transferred to a microcentrifuge tube for direct DNA extraction.

#### Grain Supplement Experiment (Experiment 2)

In a previously published experiment with a 4 × 4 latin square design, four C1-fistulated male (7 ± 1.5 years old; 61 ± 5 kg BW) alpacas were fed a series of 5 diet treatments in a random order: mixed grass hay (MGH; orchard, *Dactylis glomerata*; meadow bromegrass, *Bromopsis biebersteinii*; smooth bromegrass, *Bromus inermis*), and MGH supplemented with barley (B), amaranth (A), quinoa (Q), or soybean meal (S) (Supplementary Table S1; see amounts below) (Nilsen et al., 2015; Robinson et al., 2016). Prior to the start of the experiment, the animals were acclimated to a MGH diet, which was fed daily *ad libitum* at 0700 h for 30 days. During the acclimation period and throughout the experiment, water was also provided *ad libitum*. During the trial phase, each alpaca was fed each of the 5 diet treatments for 14 days, and no alpaca was fed the same diet twice. There was no acclimation period between diet changes. Average daily dry matter intake was measured as described above and was reported previously at 965 (0), 771 (213), 1029 (227), or 804 (218) g/d total for MGH or MGH supplemented with A, B, or Q respectively (Nilsen et al., 2015) and 887 (333) g/d for S (Robinson et al., 2016) (supplemented amount in parentheses). The supplemented grain amount was varied to normalize the protein intake, as described previously (Nilsen et al., 2015) for AB and Q, while S was fed to approximate 30% of dry matter intake. Supplementation did not significantly alter feed consumption relative to MGH alone (Nilsen et al., 2015; Robinson et al., 2016). At each sampling, the C1 pH was measured to rule out negative health effects from the supplements (e.g., acidosis) using a pH meter (Corning 340, Tewksbury, MA, United States) equipped with a combination probe. The previously reported mean pHs conferred by the different dietary supplements were above the levels associated with detrimental effects (pH 6.81, 6.66, 6.78, 6.78 and 6.65 for MGH-, A-, B-, Q- and S – fed alpacas, respectively). Feed composition, including dry matter, ash, N, and fibre, was also reported previously (Nilsen et al., 2015). Three hours post-feeding on day 14 of each treatment period, C1 samples were collected through the fistula using a rumen sampler tube (#RT Rumen Fluid Sampler Tube, Bar Diamond, Inc., Parma, ID, United States), which filtered large plant debris but permitted retention of particulates in the sample. The sampler tube was used to draw 20-mL samples of fluid from the anterocaudal, laterocaudal, and postero caudal regions of C1, which were then pooled and stored at −20°C for microbiota analysis. In the laboratory, the samples were thawed on ice, homogenized, and 250 mg were transferred to a microcentrifuge tube for DNA extraction.

#### Body Condition Experiment (Experiment 3)

Eighteen adult (∼8 years old) female alpacas were selected for C1 sampling and microbiota analysis based on an evaluation of their body condition scoring (BCS) in biannual evaluations over a 2-year period, and other characteristics: all individuals within the herd were treated the same, free of parasites, and vaccinated yearly, as well as received the same husbandry. No hierarchical competition at feed time was evident over the 2 year period, and we also selected animals that had not been reproductively active for 3 years prior to the experiment to rule out pregnancy- or lactation-dependent changes in BCS. Over a 2-year period, the BCS of each alpaca was monitored biannually. Scores were assigned by palpating the hip bones and lumbar and thoracic vertebrae of each animal to assess the animal’s fat cover (Fowler, 1998). A BCS of 1, equivalent to no fat cover, indicated very low body condition and a BCS of 5, equivalent to 5 mm fat cover, represented very high body condition. GBC and LBC animals were categorized at a score of 3 and above or 2 and below, respectively, during every evaluation. Eighteen alpacas with consistent GBC or LBC scores met these criteria. All eighteen alpacas were fed on a mixed grass pasture for spring, summer, and fall, and switched to a MGH diet for 30 days at onset of winter. The switch to MGH also reduced dietary variation with forage preferences. Because the animals were chronic GBC or LBC, feed intake was determined not to be a factor for the body condition and dry matter intake was not determined. No changes in the animals’ previous body condition were detected at the time of sampling. Material from the C1 was collected from each alpaca via oral gavage using a 9.525 mm (OD) × 2.1 m orogastric tube (Jorgenson Laboratories, Inc., Loveland, CO, United States) with an attached 60 ml catheter syringe through which 50 ml was collected to a sterile sample bottle. Samples collected included liquid and plant particulates. Between sampling of alpacas, the orogastric tube was washed and disinfected with chlorhexidine, then rinsed with water to avoid cross contamination. Samples were stored at −20°C until the sample was thawed on ice, homogenized, and collected for DNA extraction through a 50 μl wide-bore tip.

### DNA Extraction

Microbial DNA was isolated from each sample in Experiments 1 and 2 using the PowerFecal^®^DNA Isolation Kit (MO BIO Laboratories, Carlsbad, CA, United States), which includes a bead-beating step. A preliminary analysis showed no differences in the unweighted Unifrac scores of identical samples when DNA was isolated using a different kit [ZR-96 Fecal DNA Kit^TM^ (Zymo Research, Irvine, CA, United States)] that had superior economy of time and cost. For the preliminary analysis, DNA was extracted from four paired C1 samples by each of the Zymo ZR Fecal DNA MiniPrep and PowerFecal^®^DNA extraction kits. Therefore, DNA was extracted for Experiment 3 using the ZR-96 kit. Because of the difference in methods used we do not directly compare between results of the different experiments.

### PCR and Illumina Sequencing

DNA was prepared for 16S rRNA gene V4 region sequencing exactly as described previously (Kozich et al., 2013). Briefly, the V4 region of the 16S rRNA gene was amplified individually from each sample with AccuPrime *Pfx* SuperMix (Invitrogen, Carlsbad, CA) using a dual-indexing strategy as described previously (Kozich et al., 2013) (Supplementary Table S2). Samples were normalized using the SequalPrep Normalization kit (Applied Biosystems, Waltham, MA, United States). Sequencing was performed at the BYU DNA Sequencing center, and samples plus 10% PhiX control DNA were sequenced using 2x250bp v2 Illumina sequencing kits on a MiSeq (forage diet and supplement experiments, and the comparison of kit-effects), or at the BYU DNA Sequencing center on a HiSeq 2500 (body condition experiment), all following manufacturer’s recommendations. Sequences were deposited to the National Center for Biotechnological Information’s Short Read Archive under study number SRP116192.

### Sequence Analysis

Sample reads were demultiplexed on the Illumina platform and quality filtered using default parameters in QIIME 1.9.1 (Caporaso et al., 2010b). Open-reference OTU picking was performed using UCLUST (Edgar, 2010) with OTUs grouped at 97% similarity. The reads were aligned to the GreenGenes Core reference alignment (DeSantis et al., 2006) using PyNAST (Caporaso et al., 2010a). Taxonomy was assigned according to the GreenGenes taxonomy using the RDP Classifier 2.2 (Wang et al., 2007) and the GreenGenes reference base (McDonald et al., 2012; Werner et al., 2012) and a phylogenetic tree was built with FastTree 2.1.3 (Price et al., 2010). OTU tables in each experiment were filtered to exclude OTUs assigned to the Archaea. For experiment 1, diet-dependent changes in the microbiota were calculated from an OTU table that was subsampled to 5,990 reads per sample, leading to the discarding of all duodenum samples and three jejunum samples. The OTU table in experiment 2 was subsampled to 10,400 reads per sample to include all samples. In experiment 3, OTU read counts in a sample were discarded if the reagent-only controls had higher read counts and reads were subsampled to 72,000 reads per sample.

### Statistical Analysis

Statistical analyses were performed in QIIME 1.9.1 and R (R Core Team, 2016). Beta diversity was calculated using weighted and unweighted Unifrac distance (Lozupone and Knight, 2005) and differences between samples were confirmed by PERMANOVA using the R package Vegan (Oksanen et al., 2017). Differences in OTU abundance between samples were performed using the ANCOM software in R (Mandal et al., 2015).

## Results

### Survey of Alpaca Body Site- and Diet-Dependent Microbiota (Experiment 1)

**FIGURE 1:**
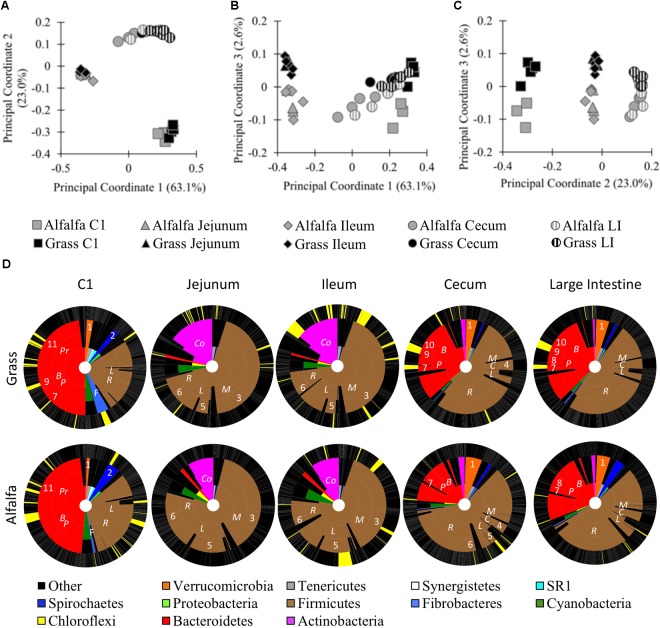
Microbial communities of the GH-fed and AH-fed alpaca digestive tracts. Principal coordinate analysis (PCoA) of samples from GH-fed and AH-fed alpacas. 16S rRNA gene sequences from alpaca C1, small intestine (jejunum, and ileum), and distal intestine (cecum and large intestine [LI]) samples were subsampled to 5,990 reads and clustered by weighted PCoA in QIIME. **(A)** Principal coordinates 1 and 2; **(B)** Principal Coordinates 1 and 3; **(C)** Principal Coordinates 2 and 3; **(D)** KRONA charts of GH-fed (top) and AH-fed (bottom) alpacas. Inner ring: Phylum levels designations. 2nd inner ring: Family-level designations, abbreviated as: *Coriobacteriaceae* (Co), *Prevotellaceae* (Pr), *Bacteroidaceae* (B), *Paraprevotellaceae* (P), *Fibrobacteraceae* (F), *Ruminococcaceae* (R), *Lachnospiraceae* (L), *Clostridiaceae* (C), *Mogibacteriaceae* (M). 3rd inner ring: Genus-level designations, abbreviated as: *Akkermansia* (1), *Treponema* (2), *Mogibacterium* (3), *Clostridium* (4), *Butyrivibrio* (5), *Ruminococcus* (6), YRC22 (7), CF231 (8), BF311 (9), *Bacteroides* (10), *Prevotella* (11). Outer ring: OTU-level designations. Yellow = significantly different abundance with a GH- or AH- diet on a body-site basis. Black = no difference in abundance with diet. Gray lines separate taxonomic designations.

To better understand how body site and diet contribute to differences in the alpaca microbiota, we performed a 16S rRNA gene survey of six sites along the digestive tract of alpacas fed GH or AH diets. One digesta sample was taken from the C1, duodenum, jejunum, ileum, cecum, and large intestine (LI) of five alpacas per treatment. A total of 1,057,086 bacterial reads were obtained on a partial Illumina MiSeq run, with an average of 15,545 reads per sample and 10,733 total OTUs We performed principal coordinate analysis (PCoA) and PERMANOVA of weighted Unifrac distances to compare the microbiota composition of different alpaca body sites in alpacas fed different diets. At a 5,990-read subsampling depth (rarefaction curve, Supplementary Figure S1A; OTU table, Supplementary Data Sheet S1), the samples grouped by body site in a PCoA (Figures 1A–C). Principal coordinates 1 (63.1%) and 2 (23.0%) separated the samples into three general locations within the digestive tract: the C1 compartment; the small intestine (jejunum and ileum); and the distal intestine (cecum and LI). PCoA analysis of unweighted Unifrac distances displayed the same trends (Supplementary Figure S2). Duodenum samples were excluded because they uniformly had few reads, but a PCoA based on shallower read subsampling that included the duodenum samples (2,400 reads/sample) showed similar trends, clustering the duodenum samples with the ileum and jejunum samples (Supplementary Figures S1B, S3 and Supplementary Data Sheet S2). PERMANOVA of the weighted Unifrac distances confirmed the visual differences in the microbiota composition between, but not within, each general location (Body site: *F*_4,44_ = 64.2, *p* ≤ 0.001; Diet x body site: *F*_4,44_ = 2.5, *p* = 0.028). Differences between communities could be attributed, in part, to the phyla dominating different sites. Diet-dependent OTU variation across the different body sites as defined by Analysis of Microbial Communities (ANCOM) revealed that most OTUs were differentially abundant with body site (Supplementary Figures S4, S5 and Supplementary Data Sheet S3). For example, in GH-fed alpacas 80.8% of reads (in 20.7% of OTUs) were differentially abundant across the five body sites (Supplementary Data Sheet S3). Rare OTUs made up most of the 6,168 OTUs that were not differentially abundant with body site: only 14 OTUs had an average abundance >5 reads/OTU (Supplementary Data Sheets S1, S3).

Principal coordinate 3 revealed sample separation by diet (Figure 1C, 2.6% of variance). PERMANOVA of weighted and unweighted Unifrac distances confirmed that diet significantly influenced the sampled microbial communities at each of the six body sites (Diet: *F*_1,44_ = 7.9, *p* = 0.002; Diet x body site: *F*_4,44_ = 2.5, *p* = 0.028, Supplementary Figure S2D). In the total dataset (body site and diet treatments combined), the amount of variance explained by PCo3 was small relative to the variance explain explained by PCo1 and PCo2, which separated samples by body site. However, the small amount of variance in the total dataset does not suggest that diet had a negligible impact on the microbiota. When we compared diet effects in each body site individually, the first principal coordinate, PCo1, visually separated samples by diet and accounted for 19.3–30.2% of sample variance (*p* < 0.05 by PERMANOVA; Supplementary Figure S6). When we examined each of the body sites independently, we also detected OTUs that were differentially abundant with diet in each body site, and no OTUs were significantly different across all body sites (Figure 1D and Supplementary Data Sheet S4). Prominent examples included differential abundance of the *Firmicutes* in all body sites; *Bacteroidetes* in the C1 and distal intestine; *Fibrobacteres* and *Spirochaetes* in the C1; and *Actinobacteria* in the small intestine (Supplementary Data Sheet S4). The OTU that significantly differed in abundance in the most body sites was a *Butyrivibrio* OTU (ID 169738) that was more abundant in the three body sites of AH-fed, relative to GH-fed alpacas (ileum, cecum, and LI). Together, these results demonstrate significant differences in relative read abundances with both body site and diet.

### Grain Supplement Effects on the C1 Microbiota (Experiment 2)

**FIGURE 2:**
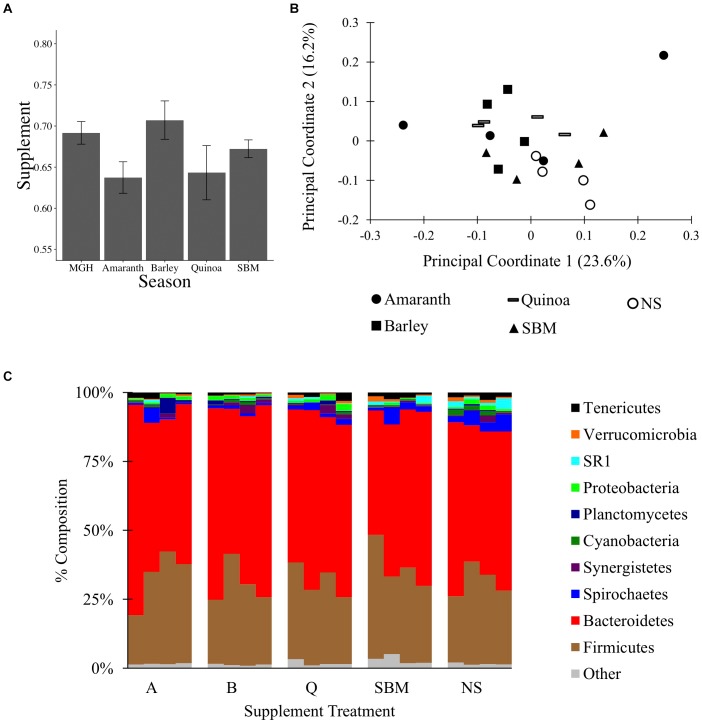
The C1 microbiota of alpacas fed mixed grass hay or mixed grass hay plus one of four supplements: amaranth, barley, quinoa, soybean meal (SBM), or no supplement (NS). **(A)** Average weighted Unifrac distances between samples and SEM. **(B)** PCoA of the C1 microbiota, performed on weighted Unifrac data from 10,400 reads subsampling depth. **(C)** Microbial composition of each sample at the phylum level, rarified to 10,400 reads per sample.

To test if MGH supplemented with grain can alter the C1 microbiota, a 16S rRNA gene survey was conducted on material from C1-fistulated male alpacas fed MGH or MGH supplemented with amaranth (A), barley (B), quinoa (Q), or soybean meal (S). The different grains were selected to compare the effects of feeding pseudo-grains common in South America (A, Q) and the US (B), and a common protein supplement (S). At the end of each 2-week period, C1 material was sampled and surveyed by 16S rRNA gene sequencing. A total of 450,617 filtered reads were obtained by Illumina MiSeq sequencing, and reads were subsampled to 10,400 reads per sample; the reagent-only control was not analyzed because it produced only 230 reads. There was no variation in the weighted Unifrac distances between samples (ANOVA of linear model: *F*_4,25_ = 2.01, *p* = 0.12, Figure 2A), and none of the four supplements significantly altered microbiota composition relative to MGH alone or to each other (Figures 2B,C, Supplementary Figures S1C, S7, and Supplementary Data Sheet S5). Overall, these results suggest that, unlike a 4-week regime using tall fescue GH and AH (experiment 1), feeding alpacas a 2-week dietary supplement together with MGH was insufficient to significantly alter their C1 microbiota composition.

### Body Condition and the C1 Microbiome (Experiment 3)

Experiments 1 and 2 revealed varying responses of the alpaca C1 microbiota to changes in diet. To test the hypothesis that the microbiota would vary with body condition of the host, we sought to identify differentially abundant read counts in the microbiota of healthy versus chronic LBC alpacas. Body condition scores of alpacas in a UT, United States herd were monitored over a 2-year period of pasture *ad libitum* foraging during spring, summer and fall, and MGH during winter. Over the 2-year period, animals that consistently displayed wide differences in their body condition despite equivalent pasture foraging time and apparently independent of hierarchical herd effects (e.g., 1 LBC animal was at the top of the herd hierarchy and some GBC animals were at the bottom) were selected for microbiota analysis. Thirty days into winter feeding, C1 samples were collected from alpacas that were chronically classified as LBC (scores ≤ 2, *N* = 12) or GBC (scores 3+ to 5, *N* = 6), and total DNA was extracted using a different method than was used for the forage diet and supplement experiments. We tested if there was kit-dependent variation in the detected microbial communities using a set of 4 paired C1 samples from which DNA was extracted by the two methods. In these samples the microbiota differed by weighted, but not unweighted, Unifrac distance analysis, suggesting some kit-dependent differences in the microbial communities, and precluding a direct comparison of these results with those of previous experiments (Supplementary Figures S1D, S8 and Supplementary Data Sheet S6).

**FIGURE 3:**
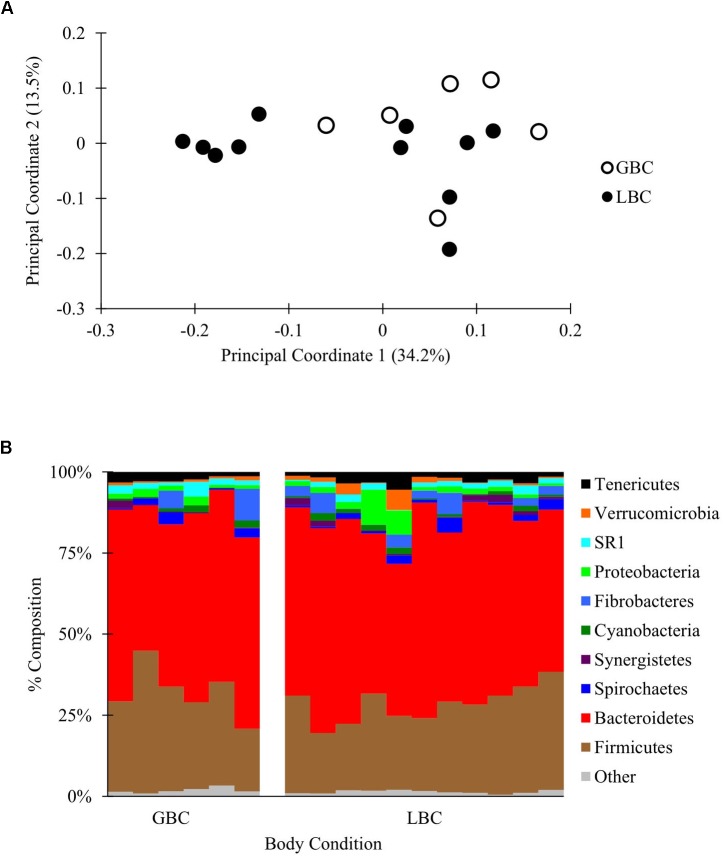
The C1 microbiota of low body condition (LBC) and good body condition (GBC) alpacas. The C1 samples from body condition-scored alpacas, subsampled to 72,000 reads/sample. **(A)** Weighted PCoA. **(B)** Taxon plot, clustered at the phylum level.

A partial lane of a HiSeq 2500 yielded 3,426,721 total reads that were filtered and subsampled to 72,000 reads per sample for beta diversity analysis (Supplementary Figure S1E and Supplementary Data Sheet S7). PCoA of weighted and unweighted Unifrac distances between samples revealed no visual or statistical clustering of the samples by body condition (PERMANOVA *p* = 0.21 for Figure 3, statistics for unweighted Unifrac in Supplementary Figure S9). Additionally, there were no differences in alpha diversity between GBC and LBC alpacas (Supplementary Table S3) or OTU abundance (by ANCOM, data not shown). Thus, we found no evidence that the presence or abundance of microbial OTUs vary with alpaca body condition.

## Discussion

In this study, our goals were to survey the alpaca digestive tract microbiota, determine how diet modifies the detected communities, and identify candidate taxa with possible influence on alpaca body condition. A survey of six body sites in GH-fed alpacas – C1, duodenum, jejunum, ileum, cecum, and LI – revealed that the different body sites clustered into three unique microbial communities – the C1, small intestine, and distal intestine. Comparisons of alpacas fed an AH or GH diet revealed significant impacts of diet on gut microbiota composition in each body site. In contrast, supplementing MGH with four different grains was insufficient to significantly alter the C1 microbiota, although this interpretation comes with the caveat of a shorter acclimation time period than in the AH/GH experiment (2 weeks versus 4 weeks). Additionally, we found no evidences of community- or OTU-level differences in the microbiota of chronic GBC and LBC alpacas from the same herd. Thus, our work reveals diet- and body site-specific alpaca microbiota but provides no evidence that microbial relative abundance or identity as defined by the V4 region of the 16S rRNA gene alone are significantly associated with alpaca body condition scores.

Results of the three experiments we performed are generally consistent with the existing literature and add novel findings about the microbiota of the alpaca small and large intestine. Rumen microbial analyses are common in studies of camelids and various ruminants (Kong et al., 2010; Pei et al., 2010, 2013; Samsudin et al., 2011; Jami and Mizrahi, 2012; Gharechahi et al., 2015; Henderson et al., 2015), but GI data beyond the rumen are sparse, with no published studies of the microbial communities in camelid intestines. The dominant phyla detected in our analyses, *Firmicutes* and *Bacteroidetes*, are abundant in existing studies of ruminants and pseudoruminants (Kong et al., 2010; Samsudin et al., 2011; Jami and Mizrahi, 2012; Mao et al., 2013, 2015; Gharechahi et al., 2015; Henderson et al., 2015), and support previous conclusions of broad similarities in the set of microbes shared between the two suborders. For example, in experiment 1, *Bacteroidetes* and *Firmicutes* were the dominant C1 phyla, representing 50.0 and 26.0% of total C1 reads with a GH diet and 48.4 and 32.7% with an AH diet, respectively. These two phyla were also abundant in the bovine rumen [40.0–50.0% and 43.0– 53.9% of reads, respectively, (Kong et al., 2010; Jami and Mizrahi, 2012)], and the dromedary camel foregut [29.6–51.2% and 30.1–61.0%, respectively (Samsudin et al., 2011; Gharechahi et al., 2015)]. The four most abundant genera in the alpaca C1 samples were *Prevotella* and unclassified genera belonging to *Clostridiales*, *Bacteroidales*, and *Ruminococcaceae*; these were also the most abundant bacterial genera in ruminant and South American camelids (Henderson et al., 2015). Although the most abundant taxa were similar among studies, differences were observed in the percent of total reads that could be attributed to each taxon. Since different sequencing methods were used in each of the studies, few comparisons can be made in the observed microbial abundances.

Experiment 1 demonstrates the presence of at least three distinct microbial communities within an individual alpaca; the C1, the small intestine (jejunum and ileum), and the distal intestine (cecum and LI). The body-site dependent distinctions are consistent with previous studies in goats, sheep and dairy cattle that showed differences in the foregut, small intestine, and large intestine or hindgut microbiota (Mao et al., 2013, 2015; Perea et al., 2017). Different nutritional functions are known for these three subsections of the digestive tract. The rumen and C1 contain microbes that ferment plant material, producing VFAs like acetate, propionate and butyrate, and are also the sites of VFA absorption, whereas the small intestine is responsible for further digestion and absorption of nutrients (Owens et al., 1986). The cecum and large intestine are sites of fermentation, VFA production, and water and electrolyte absorption (Hofmann, 1989). The presence of different microbial communities in each subsection may contribute to their physiological functions (Mao et al., 2015). For example, *Coriobacteriaceae*, which have been reported to activate polyphenols (Clavel et al., 2014), were abundant in the alpaca ileum and jejunum in this study, whereas *Prevotella*, a diverse genus of bacteria that vary in their abilities to degrade polysaccharides and proteins (Avguštin et al., 1997), were more abundant in the C1. Focusing studies on these bacteria may reveal more about the physiological processes linked to the digestive tract.

In experiment 1 we detected diet-dependent variation in the alpaca microbiota, consistent with the current understanding that diet strongly shapes mammalian GI-tract microbiota composition (Muegge et al., 2011; Carmody et al., 2015; Henderson et al., 2015). Alpacas fed either AH or GH for 30 days displayed significant differences in microbiota composition at each of the six tested body sites. Differences were most obviously attributable to site-specific shifts in *Bacteroidaceae*, *Prevotella*, and *Actinobacteria*, with additional significant differences in the less abundant taxa *Tenericutes* and SR1. In contrast, supplementing a mixed bromegrass and orchard diet with each of four different grains (experiment 2) did not influence the microbiota. However, this interpretation comes with at least two caveats. First, the grain supplements were administered for 2 weeks, as opposed to the 4-week regimen for the alpacas fed AH or GH. Second, with only one microbiome sample per animal, we cannot rule out technical noise. Together, we are unable to completely rule out that addition of minor supplements to the diet influences the microbiota in the same ways as a complete dietary shift. However, our data suggest that if there is an effect, it is smaller in magnitude than that influenced by two completely different forage diets, AH and GH.

We observed no overall differences between the C1 microbial communities of LBC and GBC alpacas in experiment 3. Since the microbiome has been associated with weight or body condition scores in numerous other animals (Turnbaugh et al., 2006; Nkrumah et al., 2008; Wang et al., 2012), we propose at least six, non-exclusive explanations why we identified diet-, but not BCS-score-, dependent variation in the microbiota. First, there may be sex-specific effects. The forage and supplement experiments were performed on samples from male alpacas, whereas it was necessary to perform the BCS experiment using female alpacas to obtain enough sex-controlled samples from alpacas with good and low body condition scores. The assumption of variation in the female alpaca C1 microbiota based on the earlier male experiments may not be appropriate, although we are unaware of any ruminant studies directly testing for a sex-specific microbiota in adult animals. Second, the sampling and DNA extraction methods differed between experiments (gastric tube live sample in the body condition experiment versus hand-collected dissection samples in the forage diet experiment), which could potentially alter the composition of the detected microbial communities. Third, our microbiota data do not account in any way for differences in nutrient uptake in the alpacas, such as through differences in host immunity, inflammation, or environment [e.g., the pH-dependent absorption of VFAs, e.g. (Dijkstra et al., 1993)]. Fourth, there may be gene content or gene expression differences between bacteria that have clustered V4 sequences (Zhu et al., 2015). Fifth, there could be genotype x microbiome interactions, where alpacas with LBC respond differently to the same microbiota (Shen et al., 2016). Finally, the C1 microbiota may not contribute to LBC, which may instead be determined by microbes from other body sites [as in lambs, e.g. (Perea et al., 2017)], or may have stronger contributions from host genotype independent of the influence of associated microorganisms. Our data do not favor one possibility over any of the others, and future experiments (e.g., metagenomic, metatranscriptomic, GWA studies) could help to address some of these gaps. The current work establishes the need for more in-depth analyses.

In summary, this study used Illumina sequencing to study the gut microbiota of alpacas fed different forages. The presence of unique microbial populations in different parts of the digestive tract under different dietary treatments suggested the alpaca microbial communities are sufficiently flexible to be modified by dietary interventions. However, the absence of any taxonomic differences between LBC and GBC alpacas suggests that body condition scores may not have a strong link to variation in the C1 microbiota. We recommend that further studies on the microbial gene activities (expression) and host genetics underlying alpaca low body condition could prove fruitful in efforts to improve the health and wellness of unproductive herd members.

## Author Contributions

CC, KO, KD-M, and TR performed the experiments. GS, TR, and JC oversaw the experiments. CC, KO, NR, and JC wrote the manuscript. All authors edited and approved the final version of the manuscript.

## Conflict of Interest Statement

The authors declare that the research was conducted in the absence of any commercial or financial relationships that could be construed as a potential conflict of interest.
